# Post-COVID-19 acute sarcopenia: physiopathology and management

**DOI:** 10.1007/s40520-021-01942-8

**Published:** 2021-07-30

**Authors:** Karolina Piotrowicz, Jerzy Gąsowski, Jean-Pierre Michel, Nicola Veronese

**Affiliations:** 1 Department of Internal Medicine and Gerontology, Faculty of Medicine, Jagiellonian University Medical College, 2 Jakubowskiego St., building I, 5th floor, 30-688 Kraków, Poland; 2 Department of Geriatrics, University of Geneva, Geneva, Switzerland; 3 Department of Internal Medicine, Geriatrics Section, University of Palermo, Palermo, Italy

**Keywords:** COVID-19, Sarcopenia, Acute sarcopenia, Rehabilitation, Older adults, Inflammaging

## Abstract

In this review, we discuss the pathophysiologic and management aspects of acute sarcopenia in relation to SARS-CoV-2 infection. COVID-19 is as a multi-organ infectious disease characterized by a severe inflammatory and highly catabolic status, influencing the deep changes in the body build, especially the amount, structure, and function of skeletal muscles which would amount to acutely developed sarcopenia. Acute sarcopenia may largely impact patients’ in-hospital prognosis as well as the vulnerability to the post-COVID-19 functional and physical deterioration. The individual outcome of the COVID-19 and the degree of muscle mass and functional loss may be influenced by multiple factors, including the patient’s general pre-infection medical and functional condition, especially in older adults. This paper gathers the information about how the SARS-CoV-2 hyper-inflammatory involvement exacerbates the immunosenescence process, enhances the endothelial damage, and due to mitochondrial dysfunction and autophagy, induces myofibrillar breakdown and muscle degradation. The aftermath of these acute and complex immunological SARS-CoV-2-related phenomena, augmented by anosmia, ageusia and altered microbiota may lead to decreased food intake and exacerbated catabolism. Moreover, the imposed physical inactivity, lock-down, quarantine or acute hospitalization with bedrest would intensify the acute sarcopenia process. All these deleterious mechanisms must be swiftly put to a check by a multidisciplinary approach including nutritional support, early physical as well cardio-pulmonary rehabilitation, and psychological support and cognitive training. The proposed holistic and early management of COVID-19 patients appears essential to minimize the disastrous functional outcomes of this disease and allow avoiding the long COVID-19 syndrome.

“So, roughly speaking,

we might say that getting COVID-19 is like packing a year’s worth of risk into a week or two”

David Spiegelhalter

*Statistician, communicator about evidence, risk, probability, chance, uncertainty, etc. *

*Chair, Winton Centre for Risk and Evidence Communication, Cambridge*.

## Introduction

The severe acute respiratory syndrome coronavirus 2 (SARS-CoV-2) infection is associated with a wide spectrum of presentations, from seemingly mild asymptomatic disease to severe acute respiratory failure requiring ventilatory support with accompanying multi-organ involvement [1]. The acute inflammatory response to the infection, which includes marked elevation of inflammatory markers up to the level of cytokine storm, has a high potential to harm a broad spectrum of organs and organ systems [2]. Clinical observation indicates that during the acute phase of infection, lasting approximately 2 weeks, the patient is at risk of losing 5–10% of body weight [3, 4]. Although dehydration and muscle loss likely contribute the most to this, the issue has not been elucidated in detail [5, 6]. The risk of acute sarcopenia and possible cachexia should be highest in older patients with coronavirus disease 2019 (COVID-19) [6, 7]. The clinical observations indicate that sarcopenia may either develop acutely within a matter of days (28 days) [8], or insidiously over the span of months (6 months) and years [9]. The majority of hitherto conducted research concentrated on sarcopenia resulting from aging and acute disease [10]. This, however, is not always equivalent to acute sarcopenia [8]. The latter has been studied in the GLISTEN study [11], and several studies currently under way aim at researching acute sarcopenia proper [12–14].

**Fig. 1:**
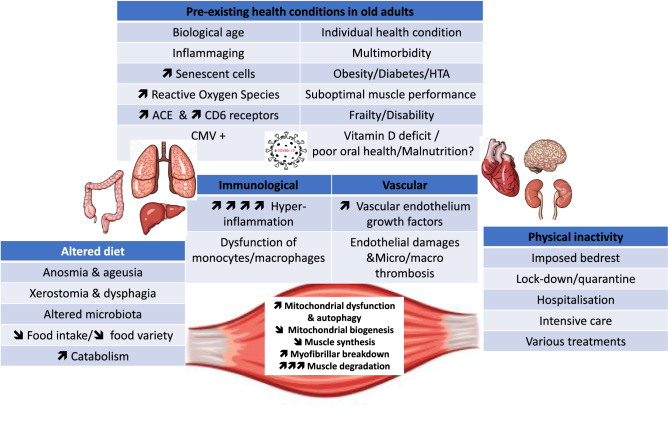
Main physiopathological mechanisms involved in the development of post-covid sarcopenia

Sarcopenia may largely impact patients’ in-hospital prognosis as well as the vulnerability to the post-COVID-19 functional and physical deterioration [15]. This may include both pathological changes in organ systems and functional deterioration in patients exemplified by the inability to cope with the daily life tasks or development of the psychologic disturbances [16]. In a study of post-COVID-19 recoverees, the strength of the biceps brachii and quadriceps femoris were 69 and 54% of the predicted normal value, in 73 and 86% of patients, respectively. The functionality of these large muscle groups was impaired likewise [17]. The individual outcome of COVID-19 and the degree of muscle mass and functional loss may be influenced by multiple factors. These, as discussed further, would include the patient’s general pre-infection medical condition, the degree of inflammatory involvement, anorexia (due to loss of appetite resulting from acute disease, anosmia and ageusia), physical inactivity, cardiovascular status, and gut microbiota. The goal of this paper is to analyze in detail the implication of these factors in the development of acute sarcopenia and try identifying the best measures to prevent and treat this acute and long-term, disabling condition (Fig. 1).

## Importance of the patient’s general pre-infection medical condition

Immunosenescence, which is characterized by elevated levels of blood inflammatory markers, entails delayed and reduced activation of innate immune response followed by an ineffective or uncoordinated adaptive immune response, which does not allow an appropriate control of SARS-CoV-2 replication [18]. Immunosenescence of the innate immune system is characterized by reduced cellular superoxide production and capability for phagocytosis. The reduced naïve-to-memory cell ratio and expansion of mature cell clones characterize immunosenescence of the acquired immune system [19, 20]. Even if the precise and intricated mechanisms are still in exploration, many physiological phenomena have been proposed to explain the immune response remodeling over time, including chronic exposure to antigens, impaired telomerase activity, mitochondrial dysfunction, defective autophagy, endoplasmic reticulum stress, defective ubiquitin–proteasome system, and age-related changes in the composition of gut microbiota [21]. Aging is also associated with the increase of the reactive oxygen species (ROS) generation in mitochondria, their vacuolization and enlargement. Age-related changes in mitochondria and mitochondrial pool add to the susceptibility to the development of sarcopenia. Muscle, as a high energy turnover tissue, possesses a large pool of mitochondria including sub-sarcolemma mitochondria (SSM, 20% of the pool), and interfibrillar mitochondria (IFM, 80% of the pool). It has been postulated that SSM are producing high amounts of ROS, whereas the IFM are more prone to autophagy [22–24]. The amount of damaged mitochondrial DNA (mtDNA) also increases, and the biogenesis of mitochondria diminishes and mitophagy increases. One of the main mechanisms involved in the regulation and dysregulation of mitochondrial quality and turnover is the mTORc1. Its inhibition is leading to decreased biogenesis of mitochondria, less production of mtDNA, more mitochondrial damage and impaired mitochondrial quality [24]. Inflammation, physical inactivity, increased adiposity, type 2 diabetes mellitus, all interact with the mTORc1 pathway, resulting in decreased muscle protein synthesis. In organisms with anabolic resistance, where exercise and ingestion of essential amino acids fails to induce adequate response in the form of mitochondrial protein synthesis (MPS) and type II fiber hypertrophy, as indicated in some studies, the mTORc1 resistance plays the key role [25].

## The SARS-CoV-2 inflammatory involvement

COVID-19 infection results in acute severe inflammation [1, 2], particularly in previously cytomegalovirus (CMV) positive patients, who exceed 80% of the population of older persons [26]. The inflammatory response may include the so-called cytokine storm, where the levels of interleukin-6 (IL-6) and tumor necrosis factor-α (TNF-α) are extremely high. The high degree of the inflammation creates a high potential for multi-organ damage, involving not only the lungs, where it leads to interstitial pneumonia and severe respiratory failure, but also the intestine, central nervous system, cardiovascular system, the kidneys and the muscle [2, 27]. The injury may be mediated by the endothelial damage and a propensity towards micro and macro thrombosis [28]. Likewise, the direct influence of inflammation is possible, with the angiotensin-converting enzyme 2 (ACE2) and cluster of differentiation 6 (CD6), as possible mediators [29, 30], although this is currently debated [31].

The acute inflammation, like the one associated with COVID-19, is a potent harming stimulus for the development of sarcopenia. Of the various harmful influences of inflammation, the elevated concentration of c-reactive protein (CRP), IL-6, and TNF-alpha, have been strongest correlates of sarcopenia and frailty [32]. One of the pivotal points in the pathogenesis and the severity of the COVID-19, with a high potential of inducing sarcopenia, is the mitochondrial damage. Ferritin, an acute phase reactant and the key player in the iron homeostasis, may directly interact with the energy production of the mitochondria [33, 34], pushing the energy production from aerobic to anaerobic modes, enhancing ROS generation and increasing cellular susceptibility to damage and cell-death.

One of the ways by which the acute inflammation of COVID-19 may augment processes leading to sarcopenia is by potentially winding-up inflammaging. Inflammaging is characterized by the increased levels of proinflammatory cytokines i.e. IL-1, IL-6, TNF-α and CRP. Inflammaging is a part of a wider spectrum of immunosenescence [19, 32]. Of the innate immune system, monocytes and macrophages are suggested to contribute to inflammaging more than any other cell type. The susceptibility to an antigenic load the reduced anti-inflammatory responses and increased proinflammatory capacity, results in progression of age-related pathologies and individual vulnerability to sarcopenia [35]. Inflammaging has been implicated in the progression of sarcopenia, frailty, and dementia [32, 36]. Especially in older subjects, the high level of inflammatory factors, as observed in COVID-19, may influence the acute changes in the body build, especially the amount, structure, and function of skeletal muscles which would summarily amount to sarcopenia [37].

## Impact of immobility and bedrest on the development of acute sarcopenia

The crucial occurrence in the train of events leading to sarcopenia is low physical activity and bedrest [6]. During the COVID-19 pandemic, the bedrest and low physical activity or bedrest could be associated either with the acute disease or with the universal imposition of lock-down measures and social distancing. It has been demonstrated that immobilization translates into significant changes of muscle cross-sectional area, volume, and mass, promoting the metabolic dysfunction (anabolic resistance) and leading to impaired functionality [25, 38]. Kortebein et al. demonstrated that a ten-day forced immobility of healthy older persons with mean age of 67.0 ± 5.0 years, would translate to 6.3% decrease of lower limb lean body mass and corresponding 15.6% decrease of isokinetic force, 14% decrease of stair-climbing force and overall 2% decrease of VO2 max [39, 40]. These results are in line with results by others [41]. However, these data predate COVID-19 era. It is possible, that due to the impact of SARS-CoV-2 infection on energy producing capabilities, the above-mentioned decreases could be even larger. Likewise, longer hospitalization times might translate to larger proportional damage to muscle, as the process may be rather exponential than linear. The immobilization during COVID-19 hospitalization differs markedly from the immobilization due to other conditions such as limb-fracture, NYHA stage IV heart failure or severe pneumonia of other causes. In COVID-19, the patient may experience profound weakness, spend hours on high-flow oxygen therapy or in the prone position, stay in the ICU which as shown by Mayer et al., was associated with a median decrease of 18.5% of rectus femoris muscle between first and seventh day of the ICU stay [42]. They may also develop the post intensive care syndrome (PICS) which apart from muscular weakness encompasses fatigue, impaired thinking, difficulty swallowing, anxiety, depression and sleep disturbances [43, 44]. Translating the results of the GLISTEN study [11] to the COVID-19, there should be a 38.4% unadjusted risk of sarcopenia associated with average length of COVID-19 associated hospital stay of 11 days [25].

Inactivity is related to marked metabolic derangement [38]. The studies of step reduction, both in younger (mean age 29 years) and older (mean age 69–72 years) healthy adults demonstrated decrease in insulin sensitivity resulting in greater area under the curve (AUC) of insulin and peptide C after glucose challenge, and greater insulin, peptide C and triglyceride AUC after the fat challenge test [45, 46]. Olsen et al. demonstrated that the amount of visceral fat increases preferentially, a finding of further metabolic consequence [45]. The changes in the glucose tolerance, but also the proinflammatory cytokines, were not reversed after 14 days of the return to previous level of step activity [38, 47]. The COVID-19 pandemic changed patterns of physical activity. The Effects of home Confinement on multiple Lifestyle Behaviors during the COVID-19 outbreak (ECLB-COVID19) was a large multi-national web-based questionnaire study that examined an impact of the lock-down measures associated with the first wave of COVID-19 in 1047 persons, 9.8% of whom were > 55 years [48]. The physical activity decreased, by approximately 1/3, from the baseline vigorous (39 min/week), moderate (32 min/week), walking (37 min/week), and all physical activity (108 min/week). The sitting time increased from 5 to 8 h per day. Of note, the baseline level of the activity in this group was significantly lower than what is generally recommended [49]. Another study presenting the data for a wide age spectrum indicate an increase of the number of hours spent in bed at night [50]. Likewise, the type of the activity changed during the pandemic, however, the data pertaining directly to older subjects are lacking.

## The impact of diet on acute sarcopenia development

An inappropriate diet is an important factor in the development of sarcopenia. From caloric content through essential amino acids, vitamins, macro, and micro-elements, dietary fibers, to hydration. It may act on several levels. During the acute stage of the COVID-19 disease, the anosmia (41.0–52.7% of COVID-19 cases) and ageusia (38.2–43.9% of COVID-19 cases) have been linked to the involvement of an array of accessory cells (like the sustentacular cells) in the olfactory epithelium which then interact with the olfactory nerve structures, or with the taste-receptor structures in the taste buds, respectively [51–53]. However, the direct Central Nervous System involvement cannot be excluded. Of note the presence of these symptoms may be less frequent in older age, due either to preceding gustatory deterioration or to the fact that these symptoms tend to be less frequent in the more severe form of COVID-19 as seen in older adults [51]. It has been hypothesized that SARS-CoV-2 virus may competitively occupy the taste-bud sites for sialic acid, depriving the taste molecules of their protection against too fast an enzymatic breakdown [53]. Within a month of the infection up to 30% of cases of olfactory dysfunction and 20% of gustatory one experience no recovery, and in those with recovery some will experience para-osmias or distorted taste [54]. The xerostomia (45.9%) and dysphagia (28.9%) have been reported as possible coexisting COVID-19 symptoms or COVID-19 aftermath [55, 56]. However, these data rarely pertain exclusively to older persons. Poor oral health should be considered likewise, with poor denture, diminished strength of masticatory muscles and tongue, and poor salivation [57]. The difficulty swallowing may be due to the presbyphagia, augmented by the sarcopenic dysphagia, which might develop acutely in COVID-19, creating a self-winding sequence of events [58]. The latter, however, needs to be studied. Together with a pro-anorectic effect of the inflammation, and hypoxia, these may contribute to a diminished intake of foodstuffs [59]. Further, the severe inflammation coupled with the tissue ischemia increase caloric demand. The persistent inappropriate feeding in the post-COVID-19 stage may also contribute to the development of sarcopenia. Finally, the nausea and diarrhea, but also frequently seen liver dysfunction experienced by some patients may further contribute to anorexia but also to diminished assimilation [60].

The lock-down measures and social distancing have had a profound effect on the quality of diet. The tendency to rely more on the canned food, flour-based food, and rice increased. A study from Poland demonstrated that during the first national lock-down, three-fourths of surveyed persons ate vegetables or fruit once a day at best, that is less than the advised 2 vegetable servings a day, and 48.7% declared they were afraid of going out to shop for food or touch the newly bought items [61]. However, these data pertain to younger population, as only 4.9% were above the age of 40 years. The data from an international survey in Europe, Asia, and North Africa, demonstrated that during the pandemic the unhealthy food was consumed more frequently [48]. This however was likewise based on the data where only 10% were older than 55 years. An Italian study showed that in a large proportion of subjects there was an increase in the consumption of home-made pizza, and home-made sweets, but a decrease of the consumption of the fresh fish. Of note, close to 70% were having at least two servings of vegetables per day [50]. The data indicate the possibility that the compulsive eating habits increased [50, 62] and that the obese persons tended to gain weight as opposed to undernourished ones who tended to lose weight further [61]. An interesting study from the Netherlands demonstrated that between 48.3 and 54.3% of the 1119 participants aged over 61 years lowered their physical activity during the pandemic, 20.3–32.4% picked-up snacking habits, while 6.9–15.1% reported habits potentially leading to undernutrition [53].

All above, may contribute to the development of sarcopenia in several ways. From simple enough fact of caloric insufficiency, through low intake of compounds essential for the maintenance of the muscle mass, to the increased intake of foodstuffs stimulating obesity and inflammation including an intramuscular one [38]. For instance, it has been observed that a decrease of intake of the food containing the essential amino acids, and especially low intake of leucin may be associated with less physiologic stimuli for the muscle mass increase. Leucin is known to activate the mTORc1 pathway enhancing biogenesis of mitochondria and muscle growth [25]. It has observed that an anabolic resistance may likewise play a role [63]. Finally, the unfavorable eating patterns adopted by some persons or communities, may promote the development of obesity, which, when coupled with the aforementioned mechanisms of inflammation and decreased mitochondria and muscle stimulating diets, and lack of physical activity may lead to sarcopenic obesity.

## The role played by microbiota in the acute sarcopenia

It has been shown that the gut microbiota are influenced by COVID-19 [64]. The gut microbiota have been postulated to be a modulator of various physiologic actions. The influence ranges from the immune system, through metabolism, cardiovascular system, central nervous system, to body composition with muscle function [65]. The microbiota may also modulate the process of inflammaging, one of the currently recognized mechanisms of aging, and thus impact aging trajectories [66, 67]. Although widely studied in animal models and in human subjects both under physiological conditions and in select pathologies, little is known about the exact impact of the interplay between the acute inflammation in COVID-19 and the gut microbiota on the subsequent inflammaging and changes in body composition and physical performance. This would include the emergence of sarcopenia and cachexia.

A study of the Chinese population from Hong-Kong demonstrated a COVID-19 related specific composition of gut microbiota [64]. The amount of *Coprobacilli, Clostridium ramosum*, and *Clostridium hathewayi* at baseline correlated with COVID-19 severity. The amount of *Faecalibacterium prausnitzii*, an anti-inflammatory bacterium, inversely correlated with the disease severity. Also, the presence of *Bacteroides dorei, Bacteroides thetaiotaomicron, Bacteroides massiliensis*, and *Bacteroides ovatus*, the bacteria downregulating expression of ACE2 in murine models, correlated inversely with SARS-CoV-2 shedding to stool of the vet patients [64].

Studies both in the animal germ-free models and humans demonstrated that gut microbes are crucial for the development, maturation, and function of the intestinal immune system and systemic immunity [68, 69]. Futhermore, the human gut-associated lymphoid tissue maintains control over the intestinal microbiota. In turn, microbial colonization of the gastrointestinal tract affects the composition of gut lymphoid tissue. Interactions between microbes, epithelium and gut lymphoid tissue are involved in modeling of the memory mechanisms of the immune system [70]. Innate responses are mediated not only by white blood cells, but also by intestinal epithelial cells that coordinate host responses by synthesizing a wide range of inflammatory mediators and transmitting signals to underlying cells in the mucosa. However, the ability of the gut to sequester the microbes or their products declines with age [66]. Those harmful products of the microflora can leak into the surrounding tissues and the circulation, fueling the already present inflammaging [37].

## The other organs involvement and their contribution to acute sarcopenia

The pathophysiologic feature of SARS-CoV-2 infection is its dependence on the ACE2 for anchoring in the surface of the target cell [71]. Then, in the respiratory epithelia, the transmembrane serine protease 2 (TMPRSS2) and the lysosomal cathepsins are largely responsible for the facilitation of the S2 spike domain expression, which in turn leads to the formation of syncytia [72]. In other tissues, where TMPRSS2 is expressed to a lesser degree, it is probably the furin that facilitates the process. Both heart and the skeletal muscle express ACE2 abundantly. This, together with the furin-dependent pathway for the viral spread, creates the basis for the possible involvement and a potential damage to the whole-body skeletal musculature during COVID-19 [29, 57].

## The effect of the cardiovascular involvement on the development of sarcopenia is complex

COVID-19 has a high potential to induce cardiovascular complications. This stems from prothrombotic status, the burden exerted by the cytokine storm, electrolyte disorders, direct damage to ACE2 expressing tissue, and hypoxemia [73]. The patient experiencing COVID-19 related stroke, myocardial infarction, heart failure or arrhythmia whether hospitalized in an ICU or a general ward, will have diminished activity that sometimes would be restricted to bedside rehabilitation sessions. Of note, hospitalization may be complicated by delirium. In its hypoactive form this would directly lead to a diminished physical activity, and in hyperactive form might require antipsychotic treatment likely to promote bedrest as well. In some patients, despite the preventive measures a pressure ulceration would develop adding to the catabolic burden and further restricting the patient’s voluntary movements. The cardiovascular involvement usually has the potential to extend into the post-COVID-19 recovery period, with the possibility of further interfering with the skeletal muscle [15, 74]. On the other hand, the COVID-19 may develop in a patient already burdened with cardiovascular disease and thus prone to the development of sarcopenia [75]. In such case the pro-sarcopenic effects of COVID-19 would act synergistically with the burden already present [76].

The effect of the pulmonary involvement on development of sarcopenia is multidirectional. The SARS-CoV-2 infection begins with the involvement of the respiratory epithelia, including the type 2 pneumocytes. [77] These tissues express ACE2 to a high degree, and they are likewise expressing the TMPRSS2 which facilitates the second step of the viral invasion. The rapidly progressing lung involvement may produce the massive viral interstitial pneumonia characterized by a widespread ground-glass infiltrates on the lung CT, and clinically associated with marked hypoxia. It has been demonstrated that the radiologic severity of the disease translates to the longer hospitalization times, which in turn would per se translate into less physical activity [78]. A recent study showed, that up to 50% of hospitalized older persons are active only for up to 30 min per day [79]. Hypoxia adversely influences a range of functions, from sensation of hunger (by stimulation of leptin production) through diminished tissue availability of oxygen with the switch from oxidative to non-oxidative forms of energy production [34, 80]. This in turn would lead to the diminished physical activity. Hypoxia interferes with the energy consuming processes. Through the regulated in development and DNA damage responses 1 protein (REDD-1) and the 5'-AMP-activated protein kinase (AMPK)-induced inhibition of the mTORc1 which in turn decreases phosphorylation of p70s6K1 and 4E-BP1. This in turn inhibits the translation of mRNA in the muscle [59]. However, these data come from studies on cancer cells under very low oxygen concentrations. Other studies, mainly in rodents, suggest that clinically relevant hypoxia might induce protein synthesis in the muscle, this however would be offset by even greater proteolysis, leading to the net decrease of the muscle mass [15]. Hypoxia is associated with greater levels of myostatin, and the switch from the IGF-1/Akt to IGF-1/ERK which stimulates myogenesis but not the differentiation in the skeletal muscle [59]. Sarcopenia is a whole-body disorder. A good example of this is the sarcopenic involvement of diaphragm and the intercostal muscles would translate to an impediment to the ventilatory function, this closing the vicious circle [80]. Thus the sarcopenic respiratory disability may be one of the aftermaths of the COVID-19 in older persons [81]. Some insights are given by studies from the pre-COVID-19 era. Patients recovering after pneumonectomy who at the time of the procedure were sarcopenic tended to be at a higher risk of needing endotracheal intubation and full ventilatory support [82]. Likewise weaning of ventilator is negatively related to the decreased muscle mass [83]. Similar relationship may occur during COVID-19.

## COVID-19 related medications

Some of the medications currently in use to treat COVID-19, have the potential to interact with skeletal muscle. Neither Remdesivir nor fresh frozen convalescent plasma seem to interact directly with skeletal muscle structure or function. Tocilizumab, a humanized antibody against IL-6 is used in patients with pronounced cytokine storm. Data from rheumatoid arthritis patients indicate that long-term treatment with tocilizumab leads to increase of muscle mass as assessed with DXA [84, 85]. However, the extent to which this might be extrapolated to older patients with COVID-19 who undergo a short course of therapy is still debatable. Parenteral steroids have been used in patients with severe disease and respiratory failure requiring oxygen therapy. Steroids are known to increase protein turnover in the skeletal muscle, thus leading to the decrease of the muscle mass and an outright wasting [86, 87]. The hypercortisolemia and bed rest act synergistically. It has been demonstrated that after 28 days, healthy young individuals would lose more muscle when restricted to bed and given hydrocortisone than with the bedrest alone [88]. Their impact on the bone and fat tissue may enhance the emergence of the obese osteosarcopenia. However, the course of therapy with steroids during COVID-19 is short. The diabetogenic effect of steroids, may in susceptible patients add to the problem of the anabolic resistance. Oxygen used in COVID-19 patients increases the oxygenation of blood thus promoting physiological energetic reactions in mitochondria of skeletal muscles. The antibiotics used to treat potential bacterial superinfection may increase the risk of sarcopenia or lead to its aggravation by causing antibiotic-related anorexia, diarrhea including due to a clostridial infection. Finally, the sedatives used in the ICU setting, may promote delirium.

A number of medications without backing in evidence has been used in the treatment of COVID-19 patients. Among these, chloroquine and hydroxycholoquine were debated for some time. The long-term use of these medications may be associated with the development of myopathy in patients with autoimmune rheumatologic disease. However, their use in COVID-19 was short-term, with little potential for long-term consequences [89].

## Post-COVID-19 syndrome

The recovery from the acute COVID-19 does not mark full return to health. The sarcopenic, cardiovascular, pulmonary, psychological and other symptoms may lag into the recovery phase [74, 90]. Further, the physiologic, psychologic, and social aftermath of the disease, framing the so-called post COVID-19 syndrome, may further negatively impact the physical activity and adversely influence the performance and quality of the muscle. One study demonstrated that 87.4% of patients had at least one symptom extending into the post-COVID-19 phase. The majority of these symptoms included dyspnea or fatigue. Only 12.6% of patients were free from any symptom. Of note, a proportion of patients had arthralgias or pain in other parts of the body [15]. The analysis including data from previous coronavirus infections indicate that the post infection physical function and fitness may be deteriorated as late as two years after the disease [91]. These factors may purely mechanistically adversely influence the physical performance and thus promote sarcopenia [92]. The emotional disorder post COVID-19 has been observed [93]. This may result in an increase of number of people with depressed mood with the negative consequences that abulia may have on physical activity [94]. Another study of Nordic population demonstrated that while over a half of the patients were free from symptoms 1.5 to 6 months post COVID-19. In some patients the symptoms, especially dyspnea, tarried, possibly translating to less physical activity [95]. The prolonged bedrest, vasculitis with a potential to baroreceptor damage, and autonomic neuropathy may contribute to the orthostatic hypotension and induce fear of falling, which may in turn again limit physical activity [96, 97]. Although some of the data and the hypotheses regard older persons directly, the bulk of knowledge comes from the general population with the predominance of younger subjects. Sleep disturbances (insomnia and poor sleep quality) and the post-traumatic stress disorder (PTSD) have been reported among the COVID-19 survivals [97, 98]. In one metanalysis, the PTSD was reported in up to 96% of COVID-19 patients as opposed to 15% in general population [97].

## COVID-19-related acute sarcopenia—towards prevention and management

Sarcopenia is characterized by a high catabolism [25]. It is therefore not surprising that malnutrition should be a common condition among older persons with COVID-19 [99]. Along these lines, if left untreated, it is also associated with a higher mortality [100], making the nutritional counseling especially important in older COVID-19 survivors [101]. This should not only include advice to increase calorie intake, but also to ensure adequate protein, vitamin, and mineral intakes [102]. Establishing protein requirements and ensuring that the protein supply be divided among all meals and snacks is of paramount importance [103]. In these patients, dietary recommendations should include oral nutritional supplements (ONS), especially when the diet alone would not be sufficient to meet the nutritional requirements posed by the developing sarcopenia [102]. The patient with the acutely developing post-COVID-19 sarcopenia may benefit from an ONS providing at least 400 kcal per day, with 30 g protein or more. Such strategy should be continued for at least 30 days [104]. The strategy should be stepped-up to 600 kcal per day in persons at a particularly high risk of malnutrition [102]. In the post-COVID-19, post-ICU patients, the high intensity ONS should be continued for over 60 days [104]. However, the risk of refeeding syndrome should be considered in the severely malnourished patients or patients who were fasting.

The link between vitamin D deficiency and COVID-19 has been postulated [105]. Vitamin D deficiency may be of a prognostic importance, especially in the severe COVID-19 cases [106, 107]. However, whether the supplementation of vitamin D might reduce the risk of a negative outcome, including in the sarcopenic patients, remains to be established. Despite that, in some countries, the authorities recommended the vitamin D supplementation as a possible preventive measure in persons with a high risk of COVID-19 [108]. In a pooled analysis of data from 30 interventional studies, vitamin D has been shown to improve, to some extent, muscle strength. The effect has been greater in persons with baseline vitamin D deficiency [109]. These results should be confirmed in future studies, that would lead to a better understanding of the prognostic role of low serum vitamin D levels and the effect of vitamin D supplementation in the acute post COVID-19 sarcopenia.

The COVID-19 in many patients leads to the fatal outcome [110]. The changing gut microbiota have been implicated in the change of the immune response, and thus might in part be associated with a greater morbidity and possibly mortality due to COVID-19. The support of the gut microbiota with probiotics could therefore improve the immunity and help fighting the SARS-CoV-2 infection [111]. The probiotics and prebiotics have been postulated to be beneficial in the frail older persons, however the conclusive evidence as regards the post COVID-19 older patients is still to be obtained [112].

The rehabilitation in the COVID-19 and post COVID-19 older patients has been earmarked as the especially important therapeutic modality [113]. Recently, a comprehensive approach to the pulmonary rehabilitation in the COVID-19 patients has been proposed [114]. This includes stratified protocols taking into account the setting and the severity of the pathological involvement. Such approaches may be of special importance as the organ-related consequences of the COVID-19, for instance respiratory or cardiovascular [115], but also psychological, are far reaching. Respiratory rehabilitation, which in a small study of six week duration has been shown to improve pulmonary function, has indeed been able to influence quality of life and reduce anxiety [116]. The rehabilitation is always a task for a multidisciplinary team. In the case of the acute sarcopenia after COVID-19, the involvement of a multidisciplinary team is crucial to foster awareness, education and support whenever required [117, 118]. An important aspect of the rehabilitation of the post-COVID-19 patients, as shown based on the data from persons who had been treated in the ICU setting, is to combine its classic forms with other approaches such as dietary intervention, and the instrumental techniques involving the neuromuscular electrical stimulation [119].

There have been a relative paucity of data concerning the physical rehabilitation in the sarcopenic post-COVID-19 persons. A Cochrane Review from September 2020 concluded that the majority of studies are not focusing on the issues of rehabilitation and the efficacy of the physiotherapeutic interventions [120], a finding in line with earlier reviews [121]. However, some data did emerge, pointing for instance to the tangible beneficiary effects of post-ICU post-COVID-19 daily 30 min multicomponent exercise program comprising resistance, endurance, and balance training [122].

The COVID-19 has been associated with the incident stroke. Therefore the modalities employed in the general post-stroke rehabilitation may be useful in a subset of post-COVID-19 patients [123] 124. A small case series proposed that in the ICU-treated COVID-19 patients who would develop focal amyotrophy possibly associated with the prone position, electrostimulation should be used early in the treatment [125]. However, whether similar approach might be effective in the acute sarcopenia after the infection should be verified in future studies.

Finally, with the COVID-19-imposed social distancing, older persons experience more loneliness [126, 127]. Therefore, as pointed out by the World Health Organization, the emotional and indeed the practical support of older persons with the daily living tasks is much advised [128]. Also, the cognitive consequences of COVID-19, with possible obvious implications for physical activity and thus sarcopenia have been pointed to [129]. Therefore the cognitive training programs may be a valid part of any post-COVID-19 rehabilitation aiming to fight sarcopenia [130, 131].

## Conclusion

The development of the COVID-19 related acute sarcopenia may adversely influence the course of the disease in the older patient, thus adding to the already high burden of the disease. This underlines the need for proper evaluation of the patient, the introduction of tailored rehabilitation and dietary approaches. As the results of further research will become available, new insights will inform better practices to recognize, evaluate and both prevent and treat acute sarcopenia of COVID-19.
